# High-fiber rye diet increases ileal excretion of energy and macronutrients compared with low-fiber wheat diet independent of meal frequency in ileostomy subjects

**DOI:** 10.3402/fnr.v57i0.18519

**Published:** 2013-12-12

**Authors:** Hanna Isaksson, Rikard Landberg, Birgitta Sundberg, Eva Lundin, Göran Hallmans, Jie-Xian Zhang, Per Tidehag, Knud Erik Bach Knudsen, Ali A. Moazzami, Per Åman

**Affiliations:** 1 Department of Food Science, Swedish University of Agricultural Sciences, Uppsala, Sweden; 2 Nutrition Research, Department of Public Health and Clinical Medicine, University of Umeå, Umeå, Sweden; 3 Department of Medical Biosciences/Pathology, University of Umeå, Umeå, Sweden; 4 Department of Animal Health and Bioscience, Research Centre Foulum, Aarhus University, Tjele, Denmark

**Keywords:** rye bread, refined wheat, meal frequency, ileal excretion, ileostomy

## Abstract

**Background:**

Whole-grain foods and cereal dietary fiber intake is associated with lower body weight. This may partly result from lower energy utilization of high-fiber diets.

**Objective:**

In the present study, the impact on ileal excretion of energy and macronutrients in response to a rye bread high-fiber diet compared to a refined wheat low-fiber diet was investigated. Furthermore, the effect of meal frequency on apparent absorption of nutrients was studied for the first time.

**Design:**

Ten participants that had undergone ileostomy consumed standardized iso-caloric diets, including low-fiber wheat bread (20 g dietary fiber per day) for 2 weeks followed by high-fiber rye bread (52 g dietary fiber per day) for 2 weeks. The diets were consumed in an ordinary (three meals per day) and a nibbling (seven meals per day) meal frequency in a cross-over design. Ileal effluents were collected during 24 h at the third day of each of the four dietary periods and analyzed for gross energy and nutrient contents.

**Results:**

The results showed that intake of rye bread high-fiber diet compared to the refined wheat low-fiber diet caused an increase in ileal excretion of energy and macronutrients. The effect was independent of meal frequency. This suggests that a high intake of rye may result in lower availability of macronutrients for small intestinal digestion and absorption. A regular intake of rye may therefore have implications for weight management.

Epidemiological evidence in different populations supports the concept that diets rich in dietary fiber are associated with a lower body mass index (kg/m^2^) and are protective against weight gain (1–4). Recent reviews conclude that body weight is inversely associated with the intake of high-fiber and whole-grain foods but not with the intake of refined-grain foods (5–7). The effect is thought to be partly mediated by an increased satiety (8) which may contribute to a lower energy intake. Another plausible explanation of the protective effect is lowered digestibility of macronutrients and thereby lowered energy value of the diet (9). Diets high in dietary fiber provide substrates for colonic fermentation, which may be of importance for satiety and weight management (10–15).

Whole-grain cereals are an important source of dietary fiber. Rye, which is used in breads and breakfast products in the Nordic and Eastern European countries, is of special interest because it contains as much as 20% dietary fiber (16). A high intake of rye fiber has been shown to increase ileal excretion of bile acids, cholesterol, nitrogen, and fat (17) and dietary fiber polysaccharide components and organic acids (18) in humans with ileostomies.

Meal frequency can alter the physiological response to foods. A recent acute study showed lower glucose and insulin concentrations in the blood during 11 h when 13 men consumed the exact same diet divided into six eating occasions instead of three (19). Improved glucose tolerance and reduced postprandial blood insulin concentrations have been reported earlier when eating frequency was shifted from 3 to 10 meals per day (20). It has also been shown that shifting daily meal frequency from 3 to 17 during 2 weeks reduced fasting total cholesterol, low-density lipoprotein cholesterol, and apolipoprotein B fasting levels (21). However, the effect of meal frequency on apparent absorption of macronutrients has previously not been studied before. Beneficial effects on metabolic parameters could partly be an effect of lower digestibility of macronutrients. Digesta escaping small intestinal digestion and absorption can be measured by the ileostomy model, hence giving information about type and amount of substrate potentially available for fermentation in the large intestine (22).

The purpose of this study was to investigate the impact on ileal excretion of energy and macronutrients in response to a rye bread high-fiber diet compared to a refined wheat low-fiber diet, as well as meal frequency. The intake of carefully standardized iso-caloric diets and the 24-h ileal excretion allowed exact comparisons between intake and output of macronutrients and energy between two diets and eating regimens in ileostomy subjects.

## Materials and methods

### Participants

Ten participants (two women, aged 34 and 51 years; and eight men, mean age 54.4 years, range 43–65 years); were recruited to the study. All participants had undergone proctocolectomy 8–29 years before the study as a result of ulcerative colitis, and had conventional well-functioning ileostomies with no sign of inflammation. All participants were in good general health based on physical examination and blood tests before the experiment. The study was approved by the Ethical Committee of the Umeå University Hospital. Details regarding the participants have been published earlier (23).

### Study design and diets

Two experimental diets were consumed during two 2-week periods. All participants started on a refined wheat low-fiber bread diet for 2 weeks, followed by the high-fiber rye bread diet for the following 2 weeks. The diets were eaten as seven meals per day (nibbling) or three meals per day (ordinary). Meal frequency was changed in the middle of the 2-week periods and arranged in a cross-over design. Participants were randomly allocated a meal frequency order (nibbling–ordinary or ordinary–nibbling) within each 2-week period. The first 2-week period was followed by a 1-week wash-out when the participants kept their ordinary diet.

Throughout the study the participants were provided with all foods constituting a standardized daily menu. Thus amounts and types of foods and drinks were identical every day during each diet period, except for the breads. The wheat low-fiber treatment included wheat-flour soft bread (142.8 g/day) and wheat crisp-bread (Wasavete^®^, Wasabröd AB, Sweden; 92.4 g/day). During the rye high-fiber treatment rye-bran soft bread (180.6 g/day) and whole-grain rye crisp-bread (Husman^®^, Wasabröd AB, Sweden, 91.0 g/day) were provided. Details about the ingredients and composition of the breads and diets as well as administration method have been described earlier (23).

Ileostomy effluent was collected on day 3 of each dietary treatment (high-fiber nibbling, high-fiber ordinary, low-fiber nibbling, and low-fiber ordinary). On that day, the participants were admitted to the research ward and stayed in a nearby hotel overnight. The ileostomy bags were changed every 2 h from 7:00 to 21:00 hours on the sampling days and at 7:00 hours on the following day. The bags were immediately frozen, and effluents from each 24-h period were freeze-dried to constant weight, mixed, homogenized, and stored at –70°C until analysis. A representative sample of the food was collected on the sampling days and treated in the same way as the ileostomy effluents.

### Chemical analyses

Before chemical analyses, samples were ground in a Cyclotec Sample Mill (Tecator AB, Sweden) to pass a 0.5-mm screen. Dry matter content was determined by oven drying at 105°C for 16 h. Results are reported on a dry matter basis and as a mean of duplicate measurements.

Crude protein (Nx6.25) was analyzed by a standard AOAC method (24) and crude fat was analyzed gravimetrically by extraction with petroleum ether after acid hydrolysis with HCl (25). Amylase-available starch (including free glucose, maltose, and malto-oligosaccharides) was analyzed by a method using a thermostable amylase (26). Dietary fiber was analyzed as the sum of non-starch polysaccharide residues, amylase-resistant starch, and Klason lignin (27). Arabinoxylan content, the main type of dietary fiber in rye, was defined as the sum of arabinose and xylose residues in the dietary fiber analysis.

The ileostomy effluents were further analyzed for content of sugar residues, including also low-molecular-weight carbohydrates and starch after direct acid hydrolysis of the effluent (28). Fructose residues in oligo- and polysaccharides which are degraded during this hydrolysis were determined separately after weak hydrolysis in 32 mM sulphuric acid (29). This procedure increases the amount of analyzed carbohydrate compared to when only amylase-available starch and dietary fiber polysaccharides by the Uppsala method are analyzed. Total carbohydrates in effluents were thus defined as the sugar residues analyzed after acid hydrolysis plus fructose residues. The concentrations of short-chain fatty acids (including acetic, propionic, and butyric acid) and lactic acid (together denoted as organic acids) in ileostomy effluent were analyzed (30).

Gross energy was determined by combustion in a bomb calorimeter (Gallenkamp Automatic Diabetic Bomb Calorimeter, Loughborough, Leicestershire, UK). From the gross energy values and nutrient contents, the proportions of energy from fat, protein, total carbohydrates, and organic acids in ileostomy effluent were calculated. The gross energy conversion factors used were: 23.6 kJ/g, 39.3 kJ/g, and 17.0 kJ/g for the heat energy of combustion of protein, fat, and total carbohydrates, respectively (31). For organic acids, a conversion factor of 17.0 kJ/g was used.

### Statistical analysis

Data are presented as mean±SD. Daily intakes of macronutrients, total dry matter, and ash are given in grams per day (g/24 h). Two-way ANOVA was performed to evaluate the effects of meal frequency (nibbling or ordinary) and diets (high-fiber or low-fiber). Participant was included as random factor in the model. All calculations were conducted with the statistical software Minitab (version 16, Minitab Inc., USA) or SAS (version 9.2, SAS Inc., USA). The level of significance was set at *p*<0.05.

## Results

Intake of macronutrients, ash, total dry matter, and gross energy per 24 h (Table 1) was very similar between the ordinary and nibbling meal frequencies within respective diets. The rye bread high-fiber diet provided higher amounts of protein, dietary fiber, total dry matter and ash, and somewhat less starch than the refined wheat low-fiber diet, with corresponding meal frequency. This was a result of the contents of the breads, because all other foods included in the diet were the same. Higher absolute amounts of macronutrients, ash, total dry matter, and gross energy were excreted after consumption of rye bread high-fiber diet than after the refined wheat low-fiber diet, with corresponding meal frequencies.

**Table 1:** Intake and ileal excretion of macronutrients (g/24 h) and gross energy (MJ/24 h) during the refined wheat low-fiber diet (LFD) and the rye bread high-fiber (HFD) diet with ordinary and nibbling meal frequencies in ileostomy subjects (*n*=10, mean±SD)

	LFD		HFD	
		
	Ordinary	Nibbling	Ordinary	Nibbling
Dry matter				
Intake	591.7±92.3	598.2±93.6	637.8±99.7	637.3±98.5
Excretion	70.5±19.9	65.1±17.2	123.3±25.7	124.4±27.6
Ash				
Intake	24.0±3.7	24.1±3.8	28.5±4.6	28.3±4.4
Excretion	10.3±3.8	9.4±3.1	14.0±3.6	14.4±4.2
Protein				
Intake	131.5±20.3	133.6±21.2	140.1±22.1	138.8±21.4
Excretion	17.6±5.9	16.1±4.9	24.1±6.6	24.6±7.4
Fat				
Intake	87.5±13.7	89.0±13.8	88.7±13.8	89.1±13.8
Excretion	5.9±3.7	5.0±2.9	7.1±3.4	7.3±3.6
Amylase-available starch				
Intake	178.6±27.8	177.8±27.7	171.8±26.8	170.5±26.4
Excretion	2.0±0.5	1.9±0.5	3.2±0.5	3.1±0.6
Dietary fiber^1^				
Intake	20.1±3.1	20.3±3.3	52.2±8.2	51.7±8.0
Excretion	16.2±3.2	15.6±3.3	43.7±7.2	44.3±7.4
Arabinoxylan^2^				
Intake	4.9±0.8	5.0±0.8	23.6±3.7	22.7±3.5
Excretion	4.9±0.9	4.6±1.0	21.4±3.6	21.7±3.9
Gross energy^3^				
Intake	13.3±1.7	13.4±1.7	13.8±1.8	14.4±1.8
Excretion	1.4±0.3	1.3±0.3	2.4±0.4	2.5±0.5

1 Dietary fiber was calculated as the sum of non-starch polysaccharide residues, amylase-resistant starch and Klason lignin.

2 Arabinoxylan was defined as the sum of arabinose and xylose residues in the dietary fiber analysis.

3 Gross energy measured by bomb calorimeter.

The ileal excretion as percentage of intake (Table 2) of dry matter, ash, protein, fat, amylase-available starch, and gross energy was significantly higher during the rye bread high-fiber diet than during the refined wheat low-fiber diet with the exception of fat with ordinary meal frequency. Higher ileostomy output occurred irrespective of meal frequency, thus dividing diets into three or seven meals had no effect on the digestibility and absorption of the diets.

**Table 2:** Ileal excretion of macronutrients and energy (% of intake, 24 h) during the refined wheat low-fiber diet (LFD) and the rye bread high-fiber (HFD) diet with ordinary and nibbling meal frequencies in ileostomy subjects (*n*=10, mean±SD)

	LFD		HFD		
		
	Ordinary	Nibbling	Ordinary	Nibbling	*p* 4
Dry matter	11.8±2.1^a^	10.8±1.9^a^	19.3±2.4^b^	19.4±2.3^b^	<0.001
Ash	42.0±10.7^a^	38.3±8.0^a^	48.5±6.7^b^	50.0±8.5^b^	<0.001
Protein	13.1±3.0^a^	12.0±2.6^a^	17.1±3.1^b^	17.5±3.4^b^	<0.001
Fat	6.5±3.4^a^	5.6±2.9^a^	7.9±3.2^b^	8.0±3.3^b^	<0.001
Amylase-available starch	1.1±0.1^a^	1.0±0.2^a^	1.8±0.1^b^	1.8±0.2^b^	<0.001
Dietary fiber^1^	80.5±7.9^a^	76.9±10.7^a^	83.9±8.3^b^	85.7±4.4^b^	<0.05
Arabinoxylan^2^	98.1±6.1^a^	93.0±11.0^a^	91.1±8.8^b^	95.4±5.8^b^	<0.05
Gross energy^3^	10.8±2.2^a^	9.8±1.9^a^	17.2±2.7^b^	17.8±2.8^b^	<0.001

1 Dietary fiber was calculated as the sum of non-starch polysaccharide residues, amylase-resistant starch and Klason lignin.

2 Arabinoxylan was defined as the sum of arabinose and xylose residues in the dietary fiber analysis.

3 Gross energy measured by bomb-calorimeter.

4 Different superscript letters indicate significant difference within row which is attributed to diet (*p*<0.05). No statistically significant effect was observed for meal frequency (*p*>0.05).

**Fig. 1:**
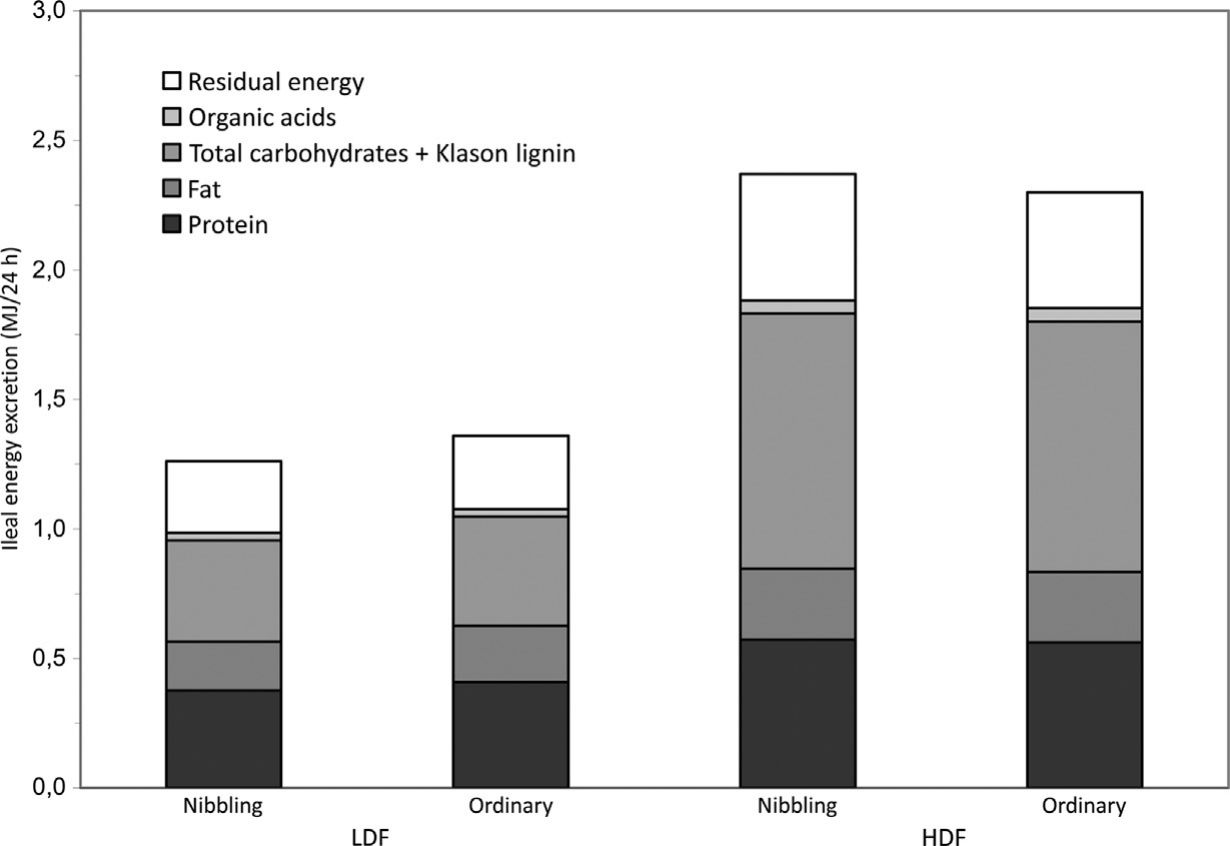
Gross energy (MJ, measured by bomb calorimeter) of the ileostomy effluents (24 h) after consumption of refined wheat low-fiber diet (LFD) and rye bread high-fiber diet (HFD) in nibbling and ordinary meal frequencies (*n*=10). Proportional contribution to the total energy content was calculated using the energy conversion factors: 23.6 kJ/g, 39.3 kJ/g, 17.0 kJ/g, and 17.0 kJ/g for the heat of combustion of protein, fat, total carbohydrates, and organic acids, respectively. Residual energy reflects the difference between total energy measured by bomb calorimeter and the calculated energy from analyzed components.

The analysis of total carbohydrates (Table 3) as described in this article as well as organic acids helped to explain approximately 85% of the dry effluent. As a mean of the two eating regimes, protein, fat, total carbohydrates plus Klason lignin, organic acids, and residual material contributed around 28, 16, 31, 2, and 23%, respectively, to excreted energy during the refined wheat low-fiber diet period, whereas the corresponding contribution to excreted energy during the rye bread high-fiber diet was 23, 12, 43, 2, and 20% (Fig. 1).

**Table 3:** Composition of ileostomy effluent (% of dry matter) during the refined wheat low-fiber diet (LFD) and the rye bread high-fiber (HFD) diet with ordinary and nibbling meal frequencies in ileostomy subjects (*n*=10, mean±SD)

	LFD		HFD		
			
	Ordinary	Nibbling	Ordinary	Nibbling	*p* 3
Ash	14.4±2.1^a^	14.3±2.0^a^	11.2±1.3^b^	11.4±1.3^b^	<0.001
Protein	24.6±1.6^a^	24.5±1.3^a^	19.3±1.5^b^	19.5±1.6^b^	<0.001
Fat	7.8±2.8^a^	7.3±2.8 ^a^	5.6±1.8^b^	5.6±1.8^b^	<0.01
Total carbohydrates^1^	34.3±4.0^a^	34.6±3.6^a^	44.0±3.0^b^	44.3±3.2^b^	<0.01
Klason lignin	0.9±0.4^a^	0.9±0.3^a^	2.2±0.4^b^	2.3±0.5^b^	<0.001
Organic acids^2^	2.4±0.7^a^	2.5±0.8^a^	2.4±0.6^a^	2.4±0.6^a^	<0.001
Total analyzed	85.1±1.9^a^	84.1±1.3^a^	84.8±1.1^b^	85.7±0.9^b^	<0.01

1 Sugar residues after acid hydrolysis plus fructose residues.

2 Include acetic, butyric, propionic and lactic acid.

3 Different superscript letters indicate significant difference within row which is attributed to diet (*p*<0.05). No statistically significant effect was observed for meal frequency (*p*>0.05).

## Discussion

Two diets including high-fiber rye breads or low-fiber wheat breads were consumed with different meal frequencies, three or seven meals per day. The high-fiber diet increased the ileal excretion of macronutrients and gross energy compared to a low-fiber diet. Despite a possibly lowered energy utilization from a rye-based diet, results from previous studies show that rye products lowered hunger ratings and increased post meal satiety compared to iso-caloric wheat bread breakfast (32, 33) up to 8 h after intake (34, 35).

Lower digestibility of the high-fiber diet provides more material that may facilitate colonic fermentation and this process may induce perceived fullness and lower the desire to eat through satiety-related signals such as GLP-1, oxyntomodulin, and PYY (10–37).

The present findings are in agreement with earlier results observing lower apparent absorption of macronutrients with high-fiber diets (9, 17, 18, 38–45). The present study also showed that this effect is independent of meal frequency. Thus eating the same diet divided into three or seven meals per day had no effect on apparent digestibility and absorption in the small intestine. Large differences in ileal excretion of gross energy measured by bomb calorimeter were found. The average ileal excretion was increased from 1.4 MJ/24 h during consumption of the refined wheat low-fiber diet to 2.4 MJ/24 h during consumption of the rye bread high-fiber diet. Gross energy reflects the maximum energy content in foods at complete *in vitro* combustion. However, not all combustible energy is available to the human body for weight maintenance (46). The energy that remains after accounting for important losses to feces, urine, and gaseous energy from microbial fermentation is referred to as metabolizable energy. An important difference between gross energy and metabolizable energy is the energy available from dietary fiber, which contains the same amount of combustible energy as other carbohydrates, but it is by definition incompletely metabolized and therefore provides less energy to the human body. To make an estimate of the energy content of the ileal effluents which can be related to hypothesized metabolizable energy – conversion factors for metabolizable energy can be used for protein, fat, and carbohydrates. The values most commonly used in dietary recommendations and for food labeling purposes are 17 kJ/g, 37 kJ/g, and 17 kJ/g for protein, fat, and available carbohydrates, respectively. Using those factors shows that the difference between the two diets is about 0.2 MJ (50 kcal) per day from protein, fat, and amylase-available starch. This difference corresponds to 2% of daily energy intake and is of relevance for public health. It has been estimated (47) that affecting energy balance by 100 kcal per day would prevent weight gain in most populations. Dietary fiber is not digestible by human enzymes and the energy content of dietary fiber is only partly available to the human body after colonic fermentation. The gross energy content of carbohydrates is 17 kJ/g. An average energy conversion factor of 8 kJ/g has been recommended for dietary fiber (48, 49). At arriving at this factor, it is assumed that 70% of dietary fiber in a total diet is fermentable (50). It is further recognized that about 60% of the energy of the fermented fiber comes out as short-chain fatty acid and that virtually all of those are taken up in the colon and available as energy for the human body. That total energy availability from a high-fiber diet is lowered is supported in animal studies, indicating a role for arabinoxylans as the inhibiting factor for feed digestibility and animal growth (51, 52).

In conclusion, this study showed that intake of a rye bread high-fiber diet compared to a refined wheat low-fiber diet decreased the apparent absorption of macronutrients from the small intestine, whereas meal frequency appeared to have no effect. Thus, a high intake of rye fiber products results in less energy available for small intestinal digestion and absorption and increased amounts of substrates made available for colonic fermentation. These effects may have implications for weight management.
